# Clinical features of toxic encephalopathy induced by 1,2-dichloroethane

**DOI:** 10.3389/ftox.2026.1683456

**Published:** 2026-02-04

**Authors:** Yuquan Chen, Yuqiang Lin, Yifan Ye, Meiwen Xie, Jiaqi Chen, Zhiqian Yang, Zhi Wang

**Affiliations:** Department of Occupational Diseases and Poisoning, Guangzhou Occupational Disease Prevention and Treatment Hospital (Guangzhou Twelfth People’s Hospital), Guangzhou, China

**Keywords:** 1,2-dichloroethane, clinical features, occupational exposure, poisoning, toxic encephalopathy

## Abstract

**Introduction:**

1,2-Dichloroethane (1,2-DCE) is a highly toxic industrial organic solvent that can cause acute toxic encephalopathy through occupational exposure, with underreported clinical data in English literature. To explore the clinical characteristics and patients’ response to supportive treatments of toxic encephalopathy caused by 1,2-DCE.

**Methods:**

Fifty-nine patients with acute 1,2-DCE poisoning admitted to the hospital from January 2009 to December 2022 were selected. Patients were divided into three groups based on clinical manifestations: intracranial hypertension (Group A), limb tremors (Group B), and behavioral changes (Group C).

**Results:**

Toxicology testing found that 1,2-DCE was difficult to detect in serum after more than 24 h. Of the 59 patients, 45 (76.27%) achieved complete recovery, 10 (16.95%) achieved partial recovery, and 4 (6.78%) died. Statistical analysis showed a significant difference in recovery rates among the three groups (χ^2^ = 10.612, P < 0.05). There were no statistically significant differences in symptom and cranial imaging recovery times between the three groups.

**Conclusion:**

Acute 1,2-DCE poisoning can cause severe toxic encephalopathy. Early and prolonged treatment with dehydrating agents and glucocorticoids is effective in improving prognosis, and patients with intracranial hypertension are at higher risk of death due to brain herniation.

## 1 Introduction

1,2-Dichloroethane (1,2-DCE) is a halogenate aliphatic hydrocarbon commonly used as an industrial organic solvent (Salovsky et al., 2002), this compound is a highly toxic oily liquid that has no color, but possesses volatility and emits a distinct odor similar to that of chloroform. Its primary applications include the synthesis of chemical raw materials, utilization as an industrial solvent, degreaser, metal cleaner, and adhesive agent which used widely in manufacturing industries (Yin et al., 2018). And its usage has been associated with the development of acute toxic encephalopathy caused by 1,2-DCE exposure (Chen et al., 2015). Due to its high vapor pressure, it evaporates rapidly from water, making respiratory tract absorption the primary route of exposure (Zhang et al., 2011). Inhalation of concentrated 1,2-DCE vapor has been shown to have detrimental effects on the human nervous system. Animal studies have also demonstrated that exposure to 1,2-DCE can lead to similar nerve damage diseases (Hotchkiss et al., 2010). Despite the existence of animal studies, there is a limited amount of literature available in English regarding acute 1,2-dichloroethane poisoning in humans. Moreover, such a large number of cases were exceedingly scarce and it is highly significant to report in the English literature.

Guangzhou, a fast-growing coastal city in southern China, has experienced an increase in the number of cases of acute 1,2-DCE poisoning in recent years. The initial symptoms of acute 1,2-DCE poisoning are not easily recognizable, and they vary from person to person. Guangzhou occupational disease prevention and treatment hospital is a major medical center in the region where there are many manufacturing factories. The aim of this study is to analyze the clinical manifestations and patients’ response to supportive treatments of acute 1,2-DCE-induced toxic encephalopathy. As one of the largest single-center cohorts of 1,2-DCE-induced toxic encephalopathy reported to date, this study fills the gap of large-scale clinical data in English literature, and we aim to share clinical experience to improve the standard of diagnosis and treatment globally. Ultimately, our goal is to share this knowledge and experience with the medical fraternity in order to elevate the standard of clinical treatment for acute 1,2-DCE poisoning.

## 2 Methods

### 2.1 General materials

Fifty-nine patients with acute 1,2-DCE poisoning who developed toxic encephalopathy and were admitted to Guangzhou Occupational Disease Prevention and Treatment Hospital from January 2009 to December 2022 were selected as research objects. They were divided into three groups based on prominent clinical manifestations. Data on gender, age of onset, intracranial pressure (ICP), electroencephalogram (EEG), cranial computed tomography (CT) scan and treatment response were recorded. Cranial CT imaging revealed diffuse brain tissue edema in all patients. After conducting an in-depth on-site investigation, it was discovered that a significant factor linking all the patients was their exposure to air contaminated with 1,2-DCE in their respective workplaces. Some patients were tested for blood toxins at the time of their initial consultation. Notably, all patients experienced the onset of symptoms during the winter season, which can be attributed to the practice of sealing off the doors and windows and deactivating the ventilation systems to retain heat within the workplaces, this created an environment conducive to the accumulation of high concentrations of 1,2-DCE in the materials or areas within the workplaces. As a result of this exposure. They were selected from all patients with acute 1,2-DCE poisoning admitted to the hospital during January 2009–December 2022, and all developed toxic encephalopathy.

We describe the information such as gender, age, duration of exposure to 1,2-DCE, clinical manifestations, as well as cranial computed tomography (CT) findings and the therapeutic effect observed in each case. Additionally, lumbar puncture was performed and the results are included in our analysis. Patients were classified into three groups based on pre-defined objective criteria for prominent clinical features: Group A (n = 29): Intracranial hypertension (confirmed by invasive ICP measurement via lumbar puncture, the clinical gold standard) as the primary manifestation; Group B (n = 20): Limb tremors as the primary manifestation; Group C (n = 10): Behavioral changes as the primary manifestation. For patients with overlapping symptoms, the primary manifestation was determined by the symptom that required priority intervention or was most severe at admission.

We closely observed the symptoms and signs of all patients in a dynamic manner to gain a comprehensive understanding of their condition.

### 2.2 Ethics statement

This retrospective study was approved by the Medical Ethics Committee of Guangzhou Occupational Disease Prevention and Treatment Hospital (Guangzhou Twelfth People’s Hospital). To ensure patient privacy and confidentiality, all names in the medical records were concealed and all information was securely protected. Only the investigators involved in the study had access to the recorded information and data. Written informed consent for publication was obtained from all individual participants included in the study.

### 2.3 Statistical methodology

Data were presented as means ± standard deviation. We employed a chi-square test, a nonparametric test, and ANOVA to analyze the indicators of different variables. The statistical analysis was performed using the SPSS software (Version 26.0), (power: 90%) P values <0.05 were regarded as statistically significant. Statistical power analysis was conducted for non-significant comparisons, with power values reported.

### 2.4 Treatment methods

For lowering cranial pressure, patients were treated with mannitol injection (25–50 g) combined with dexamethasone injection (5 mg) via intravenous drip, with a frequency ranging from once every 6 h to once daily. Additionally, 25% glycerol fructose injection was administered via intravenous drip once every 12 h to once daily, and furosemide injection (20 mg) was given via intravenous push once every 8 h to once daily. These dehydration drugs could be used in combination or alone, and albumin colloid was added for dehydration support when necessary. The dehydration regimen was adjusted according to cerebrospinal fluid levels and the severity of cerebral edema, with the dosage gradually reduced until discontinuation.

Hyperbaric oxygen therapy (HBOT) was initiated after patients’ symptoms were slightly stabilized, with a frequency of once daily and 10 days as one course of treatment. Strict exclusion criteria were applied to rule out patients with contraindications to HBOT.

For nutritional nerve support, ganglioside injection (80–200 mg), oracetam injection (4.0 g), and cytochrome C injection (30 mg) were each administered via intravenous drip once daily.

Sedative, anti-epileptic, and anti-arrhythmic drugs were prescribed as needed based on patients’ symptoms, including sodium valproate, clonazepam, diazepam, and maltrexone hydrochloride.

Anti-free radical therapy was conducted with reduced glutathione injection (1.8 g) via intravenous drip once daily.

Regarding surgery, decompressive craniectomy was performed in a timely manner for four patients who developed brain herniation, aiming to promptly alleviate intracranial pressure and protect brain tissue.

## 3 Results

Fifty-nine patients, including 31 (52.54%) males and 28 (47.46%) females, participated in the study. Their age) was [M (*P*_*25*_, *P*_*75*_) 24 (17, 34) years, ranging from 15 to 47 years. The working age of the patients was [M *(P*_*25*_, *P*_*75*_)] 5 (3, 15) months, All 59 patients had a history of 1,2-DCE exposure with a subacute onset of symptoms, including nausea and vomiting, headache, dizziness, generalized tonicoclonic seizures, generalized myoclonus, recent amnesia, loss of consciousness, behavioral changes, and dysarthria which were shown in Table 1.

**TABLE 1:** Clinical characteristics of 1,2-dichloroethane poisoning patients (n = 59).

Group	Number of patients								
​	Dizziness and headache	Nausea and vomiting	Limb/muscular spasm	Disturbances of consciousness	Diminished response	Psycho-logical disturbances	Decline of computing power and memory	Limb tremors	Speech disturbances
A (n = 29)	29	18	7	11	24	9	24	9	3
B (n = 20)	18	8	14	6	13	2	14	20	3
C (n = 10)	9	5	3	5	10	10	10	4	3
Total (n = 59)	56	31	24	22	47	21	48	33	9

A, intracranial hypertensive; B, limb tremors; C, mental/behavioral disorders.

For the entire cohort of patients, 12 (20.39%) cases underwent blood organic solvent volatility testing, as this group was the first to be admitted to our hospital after the onset of illness. Seven of the patients were sent for testing within 24 h of the onset of illness, and their results were positive, while the other five patients were sent for testing more than 24 h after the onset of illness, and their results were negative. As illustrated in Table 2, acetone, dichloromethane, and carbon disulfide were detected in various patients.

**TABLE 2:** Blood toxin test results in 12 patients.

Blood sampling time	Test results			
	1,2-Dichloroethane	Acetone	Carbon disulfide	Dichloromethane
<24 h (n = 7)	7	3	0	0
24 h–72 h (n = 5)	0	5	1	1
Total (n = 12)	7 (58.33%)	8 (66.67%)	1 (8.33%)	1 (8.33%)

Following prolonged treatment with steroids and/or mannitol, 10 (16.95%) patients demonstrated partial recovery, while 45 (76.27%) patients exhibited complete recovery. However, 4 (6.78%) patients succumbed to sudden cerebral hernia, despite the administration of treatment (see Table 3). The study revealed that Group C exhibited a higher incidence of complete remission in comparison to Groups A and B. Conversely, Group B demonstrated a higher rate of improvement when contrasted with Groups A and C. Notably, Group A exhibited the highest mortality rate. A statistically significant discrepancy in recovery outcomes was observed among the study groups (χ^2^ = 10.612, P < 0.05).

**TABLE 3:** Recovery of different groups.

Group	Outcome		
	Complete recovery	Partial recovery	Mortality
A (n = 29)	23 (79.31%)	2 (6.90%)	4 (13.79%)
B (n = 20)	13 (65.00%)	7 (35.00%)	0 (0.00%)
C (n = 10)	9 (90.00%)	1 (10.00%)	0 (0.00%)
Total (n = 59)	45 (76.27%)	10 (16.95%)	4 (6.78%)

A, intracranial hypertensive; B, limb tremors; C, mental/behavioral disorders.

**FIGURE 1:**
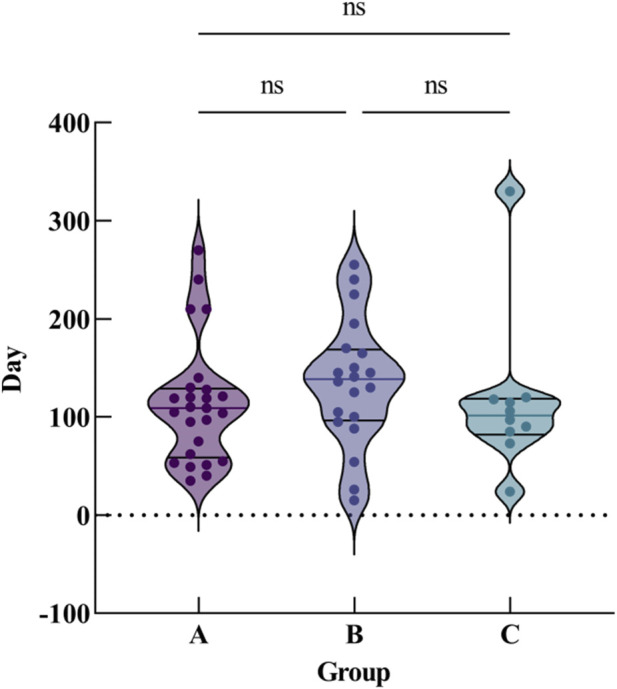
Comparison of symptom recovery time among the three groups. Group A: intracranial hypertension; Group B: limb tremors; Group C: behavioral changes. The violin plot shows the distribution of recovery days; ns indicates no statistically significant difference (P > 0.05).

Patients with 1,2-DCE poisoning in group B had a longer recovery period (135.25 ± 64.32 days) compared to groups A (119.07 ± 63.11 days) and C (115.80 ± 80.43 days). There was no statistically significant difference in the symptom recovery time between the groups (*F* = 0.630, *P* > 0.05) (Figure 1). Due to the retrospective nature of this study and the limitations of early clinical data recording standards, we did not preset *post hoc* analysis protocols during study design, and the available data mainly consist of group-level summary results, which prevents formal pairwise *post hoc* comparisons to identify specific differences between subgroups. Descriptive analysis of the summary data shows that Group C (behavioral changes) had the highest complete recovery rate (90.00%), Group A (intracranial hypertension) had the lowest complete recovery rate (79.31%) and the only mortality, while Group B (limb tremors) had the highest partial recovery rate (35.00%). This trend suggests that clinical manifestations may be associated with prognostic differences, which is consistent with the clinical significance of 1,2-DCE-induced central nervous system injury. Laboratory results showed elevated alanine aminotransferase levels in 10 patients (16.95%) (range 40.3–90.5 U/L), and all patients had normal kidney function. EEG examination was performed on all 59 patients, and 35 patients (59.32%) showed abnormal EEG, of which 25 patients (42.37%) showed slightly abnormal EEG, manifested as decreased α wave and increased δ wave, while another 10 patients (37.04%) showed moderately to severely abnormal EEG, manifested as disappearance of α wave and increased θ and δ waves. The average time for normalization of the EEG was (81.91 ± 31.25) days, with a range of 14–139 days. All patients underwent a lumbar puncture at least one time. 29 (49.15%) showed abnormal intracranial pressure (ICP). The average ICP was (262.58 ± 68.62) mmH_2_O, ranging from 18–330 mmH_2_O. Some patients presented with intractable intracranial hypertension, with intracranial pressure elevated for more than 9 months. The average time for the restoration of normal ICP was (120.11 ± 105.23) days, ranging from 10 to 280 days. Of the 59 patients, 25 (42.37%) received fundus examination, of which 8 (13.56%) patients were found to have papilledema, and the papilledema gradually recovered with the decrease of intracranial pressure, the longest recovery time was up to 97 days.

**FIGURE 2:**
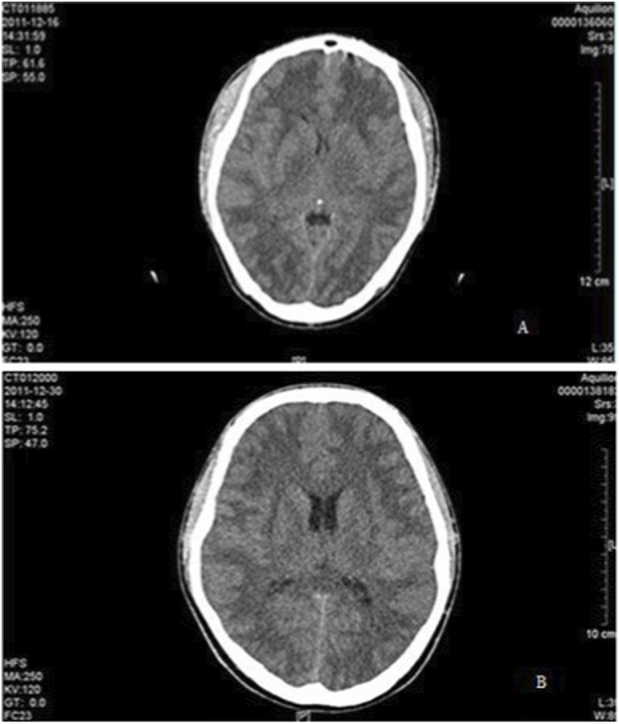
Comparison of symptom recovery time among the three groups. Group A: intracranial hypertension; Group B: limb tremors; Group C: behavioral changes. The violin plot shows the distribution of recovery days; ns indicates no statistically significant difference (*P* > 0.05).

**FIGURE 3:**
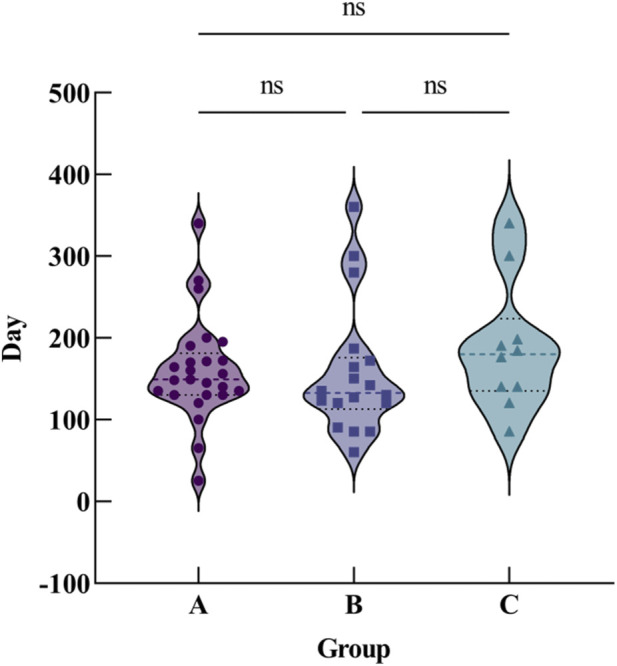
Comparison of cranial CT imaging lesion recovery time among the three groups. The violin plot displays the duration of imaging changes (in days) for Group A, Group B, and Group C. ns indicates no statistically significant difference (P > 0.05).

Based on the findings from cranial CT imaging, it was observed that the patient had a significant amount of cytotoxic edema and vasogenic edema in several areas of the brain, including the bilateral globus pallidus, subcortical white matter, bilateral nuclei dentatus, and corpus callosum (Figure 2). Recovery timefor imaging changes in this toxic encephalopathy was approximately (158.95 ± 78.25) days, with some patients taking as little as 3 weeks to recover while others required up to 1 year for complete resolution. These findings indicate that the prognosis and recovery of toxic encephalopathy can vary greatly among individuals. The duration of cranial CT imaging lesions was longer in patients with 1,2-dichloroethane poisoning, with a mean of (160.01 ± 63.22) days in group A, (157.22 ± 79.89) days in group B, and (187.30 ± 78.70) days in group C. (*F* = 0.639, *P* > 0.05) (Figure 3).

## 4 Discussion

Despite the prevalence of 1,2-DCE poisoning incidents in China, documentation of these incidents in English medical literature is sparse. Extensive research on animal models has highlighted the harmful effects of 1,2-DCE on various organ systems, including the central nervous system (CNS) (Garrison and Leadingham, 1954), respiratory system (Salovsky et al., 2002), and to a lesser extent, the kidney and liver (Cottalasso et al., 1995). Our findings, however, indicate a less pronounced manifestation of hepatic injury in cases of acute 1,2-DCE poisoning, with minor disruptions in liver function (ranging from 40.3 to 90.5 U/L) observed in a limited cohort of 10 patients who exhibited swift recovery. Renal function remained unaffected in all patients studied.

Notably, the CNS bears the brunt of 1,2-DCE toxicity, owing primarily to its high lipid solubility, which facilitates its accumulation in lipid-dense tissues like the brain. Prolonged or excessive exposure to this chemical can inflict irreversible damage on the CNS. The occurrence of 1,2-DCE-induced toxic encephalopathy is infrequently reported in developed Western nations, with only scattered case reports emerging from certain developing countries. The scarcity of comprehensive human case reports on 1,2-DCE poisoning underscores the need for heightened awareness and further research in this area.

Our study revealed that 1,2-DCE exhibits a narrow temporal window for detection within the human body, suggesting its relatively swift metabolism. Consequently, diagnostic tests conducted beyond 24 h post-exposure are highly likely to yield negative results. This narrow diagnostic window poses a major clinical challenge—for suspected cases, negative blood tests >24 h post-exposure cannot rule out 1,2-DCE poisoning, and diagnosis must rely on occupational exposure history and clinical manifestations to avoid misdiagnosis. Recovery time for imaging changes in this toxic encephalopathy was approximately (158.95 ± 78.25) days, with some patients taking as little as 3 weeks to recover while others required up to 1 year for complete resolution. This prolonged radiological recovery period suggests persistent neurotoxic damage from 1,2-DCE; even with favorable clinical recovery, long-term follow-up is necessary to monitor for potential long-term CNS sequelae. Notably, acetone was identified in the blood toxicology screens of 8 out of 12 patients, which is unsurprising given its widespread use as an organic solvent capable of dissolving adhesives. The presence of acetone in the blood samples is attributed to its presence in the raw materials, rather than indicative of an independent toxic exposure.

Prompt identification of 1,2-DCE components in the blood of patients experiencing acute 1,2-DCE poisoning is crucial for an early etiological diagnosis and an accurate assessment of the patient’s condition. This is especially important in cases of suspected acute 1,2-DCE poisoning because swift, reliable toxicological testing can provide valuable information about the poisoning in its earliest stages. This allows for timely intervention and treatment. Depending on the concentrations, 1,2-DCE poisoning can lead to various clinical manifestations. Individuals exposed to low concentrations of 1,2-DCE may experience central nervous system (CNS) symptoms similar to those caused by anesthesia. These symptoms include dizziness, headache, nausea, vomiting, weakness, tremor, unsteady walking, forgetfulness, and apathy. However, at high concentrations, the symptoms can be more severe and include central nervous system (CNS) depression. This can lead to coma, respiratory depression, delirium, tonic seizures, and even death (Zhang et al., 2011; Deng et al., 2014; Zhou et al., 2009; Nouchi et al., 1984; Liu et al., 2010). We classified all patients into three groups due to their main clinical manifestations: (A) intracranial hypertension, (B) limb tremors and (C) mental and behavioral disorders. After assessing the outcomes, it was found that group A, who suffered from intracranial hypertension, was more at risk than the other two groups, with 4 deaths reported. This highlights the importance of being vigilant for the development of cerebral hernia in affected individuals. Further analysis of the mechanism behind CNS damage revealed that previous studies had identified a potential etiological pathway involving vasogenic and cytotoxic brain edema caused by damage to specific brain regions, including the alba, globus pallidus, cerebellar dentate nucleus, and cerebral ganglia (Qiao et al., 2005; Lai et al., 2011; Xun et al., 2002). Patients suffered as a result of mental and behavioral disorder (group A) may have resulted in injury of white matter fiber tracts, which are responsible for higher brain functions (Prins et al., 2004). Furthermore, animal studies reported changes in the structure of mitochondria and the Golgi apparatus after 1,2-DCE poisoning (Bowler et al., 2003).

The clinical manifestations of toxic encephalopathy resulting from exposure to 1,2-dichloroethane are notably pronounced, exhibiting a broad spectrum of symptoms that vary according to the location and severity of the affected area. This condition predominantly impacts the central nervous system and gives rise to a diverse array of symptoms, including recurrent epileptic seizures, persistent limb tremors, and neurological abnormalities. These symptoms highlight the complexity of 1,2-dichloroethane-induced toxic encephalopathy. Patients often experience a gradual and protracted recovery from symptomatic manifestations and intracranial lesions, and the prognosis is favorable for most cases.

Most patients with 1,2-DCE poisoning experience reversible neurological damage, and the prognosis is good. However, the mortality rate is high in severe cases, primarily due to brain herniation. Currently, there are no specific drugs for treating this disease. The cornerstone of treatment involves administering dehydration medications, sedatives, and steroid therapies. Furthermore, hyperbaric oxygen therapy (HBOT) is administered with rigorous exclusion criteria for patients with contraindications. HBOT can improve the hypoxic state and reduce the inflammatory response after brain injury by inhibiting the generation of oxygen free radicals and EAAs. It also plays an auxiliary role in disease recovery. In emergent situations involving cerebral herniation, patients undergo decompressive craniectomy to promptly alleviate intracranial pressure and protect brain tissue. Remarkably, most patients demonstrated substantial improvement and achieved successful outcomes. It is important to emphasize that monitoring cerebrospinal fluid levels and adjusting the treatment regimen accordingly plays a crucial role in managing cerebral edema.

## 5 Conclusion

Acute 1,2-DCE poisoning can cause severe toxic encephalopathy, with the CNS as the primary target organ. The narrow detection window of 1,2-DCE in blood (>24 h post-exposure undetectable) complicates early diagnosis. Patients with intracranial hypertension are at higher risk of death due to brain herniation. Early initiation of supportive treatments (dehydrating agents, glucocorticoids, HBOT) and close monitoring of ICP can improve prognosis. This study, as one of the largest single-center cohorts, provides valuable clinical data for the diagnosis and treatment of 1,2-DCE-induced toxic encephalopathy, filling a gap in English literature.

## Data Availability

The original contributions presented in the study are included in the article/supplementary material, further inquiries can be directed to the corresponding author.
